# The influence of cross-regional medical treatment on total medical expenses, medical insurance payments, and out-of-pocket expenses of patients with malignant tumors in Chinese low-income areas

**DOI:** 10.1186/s12962-022-00368-x

**Published:** 2022-07-21

**Authors:** Bokai Zhang, Haixin Wang, Hongyu Zhang, Guomei Tian, Ting Zhang, Qi Shi, Jian Liu, Jinpeng Xu, Jingchu Liu, Qunhong Wu, Zheng Kang

**Affiliations:** 1 School of Health Management, Harbin Medical University, Harbin, 150081 China; 2 Department of Nuclear Medicine, The Fourth Affiliated Hospital of Harbin Medical University, Harbin, 150001 China; 3 School of Public Health, Harbin Medical University, Harbin, 150081 China

**Keywords:** Cross-regional, Patient, Out-of-pocket expense, China

## Abstract

**Background:**

In recent years, due to the increasing number of cross-regional medical patients, countries around the world have issued a series of policies or regulations to reduce their out-of-pocket burden. In this context, this study intended to explore the impact of the Spatio-temporal characteristics of cross-regional medical treatment on total medical expenses, medical insurance payments, and out-of-pocket expenses of patients with malignant tumors in low-income areas.

**Methods:**

This study included 54,904 data of cross-provincial medical treatment of malignant tumor patients insured in Heilongjiang Province, China in 2020. Firstly, Microsoft Excel 2019 and ArcGIS 10.2 were applied to conduct a descriptive analysis of the Spatio-temporal characteristics of their cross-provincial medical treatment. Then, binary and multivariate logistic regression models were used to explore the specific impact of economic level and geographical distance of medical regions on total medical expenses, medical insurance payments, and out-of-pocket expenses.

**Results:**

The number of cross-regional medical patients showed a gradual upward trend from February to December, and fell back in January. They were concentrated in regions with high economic level and short distance from the insured region, where were more likely to form the group with high out-of-pocket expenses (AOR = 3.620, *P* < 0.001; AOR = 1.882, *P* < 0.001). While this possibility in middle-distance medical regions were less (AOR = 0.545, *P* < 0.001). Afterwards, two sensitivity analysis methods showed that the results were robust.

**Conclusion:**

The number of cross-regional medical patients with malignant tumors in low-income areas is affected by seasonal factors, meanwhile, their total medical expenses, actual medical insurance payment levels, and out-of-pocket expenses are all affected by the economic level and geographical distance of medical regions. And the middle-distance medical regions may be the best choice for patients with planned cross-regional medical treatment. These provide some evidence for policymakers to improve the fairness and sustainability of medical security for cross-regional medical patients and reduce their direct economic burden of disease.

**Supplementary Information:**

The online version contains supplementary material available at 10.1186/s12962-022-00368-x.

## Background

In recent years, there have been increasing studies on universal health coverage, and the coverage rate of medical insurance has also improved worldwide [1, 2]. However, there are still some differences in the payment level of medical insurance between countries or regions [3, 4]. This is related to the influence of local political, economic, demographic, and other factors on medical insurance policies, which makes it unable to achieve uniform financing and payment standards [5, 6]. When the insured goes beyond the insured region of medical treatment, their compensation policies will change, which may be the change of reimbursement catalogue or reimbursement ratio, or even they cannot get compensation. Therefore, this study introduced the concept of trans-regional medical treatment, that is, the insured go to regions other than the insured region for medical treatment, where the insured region refers to the maximum scope of the insured's medical insurance payment policies that will not change, which can be a city, province, state, or even a country, for example, the English National Health Service (NHS) [7]. In the European Union (EU) and China, although there are European Health Insurance Card (EHIC) and universal health insurance plan respectively, due to the differences in social health insurance policies between member states or provinces, neither of them is fully integrated [8–10]. Therefore, the cross-border medical treatment in Europe is similar to the trans-provincial medical treatment in China, both of which are common cross-regional medical treatments.

Since the twenty-first century, relevant survey results show that the demand for cross-regional medical treatment is increasing [11, 12]. There may be two reasons for this. On the one hand, with the global economic development, cultural integration, and climate change, more and more people choose to work, study or settle down in other regions. The concept of a global village has gradually become a reality, resulting in the continuous increase of population migration and mobility [13–16]. It is worth noting that, although both refer to the geographical or spatial movement of population between two regions, the former usually involves a permanent change in residence or nationality, which naturally leads to a change in the insured region [17]. The latter is related to China’s household registration system, which generally refers to the separation of households, and their residence temporarily changes, but their household registration and insured region remain unchanged [18], such as migrant workers, office workers on business trips, and “migratory birds” population. According to the definition of cross-regional medical treatment in this study, people whose regions of residence are different from their insured regions form the first type of cross-regional medical treatment group when they seek medical treatment in their regions of residence, which can be called unplanned cross-regional medical treatment. On the other hand, due to the uneven distribution of medical resources and the increasing health needs of people, especially in the border areas or poor areas, where medical resources are relatively backward, people choose planned cross-regional medical treatment to seek better medical services [12, 19], such as medical travel and remote referral [20, 21], these fall into the second category known as planned cross-regional medical treatment.

A series of policies and regulations on cross-regional medical insurance payments have been issued to safeguard cross-regional patients' right to equal access to medical security and relieve their pressure to pay medical expenses in advance. Typical examples include the Regulation (European Community) 883/2004 and the Directive 2011/24/EU of the European Parliament and the Council on the coordination of social security systems [22, 23], and the policy of direct settlement of cross-provincial social medical insurance [24]. They all divide the cross-regional medical treatment into planned and unplanned, and can realize the payment method of the instant medical insurance settlement, but there are differences in the provisions of the insured's authorization, reimbursement scope, and reimbursement ratio. The advantage of the EU's cross-border medical treatment policy is that emergency patients who do not plan to seek medical treatment can directly receive medical insurance payments without prior authorization. In addition, due to the different payment principles stipulated in Regulation 883/2004 and the Directive 2011/24, patients can choose the payment path that maximizes the medical insurance compensation according to the actual situation of the inflow country and outflow country reducing the differences in out-of-pocket expenditure caused by the differences in medical service price or reimbursement ratio between insured regions and regions of medical treatment [21]. However, Chinese policies require that all patients seeking medical care across provinces obtain prior authorization for direct settlement, and unify requirements of medicare payments according to lists of medical insurance of regions of medical treatment and reimbursement rate of insured regions [21]. This has sever limitations, resulting in unavoidable treatment differences between patients in the same insured region due to different lists of medical insurance of regions of medical treatment [6]. In addition, due to widespread local protectionism, the health care sector has reduced the proportion of payments for some planned cross-regional medical treatment in an attempt to curb the outflow of patients, further exacerbating the differences [25, 26].

At present, the research on cross-regional medical insurance payment mainly focuses on the macro impact evaluation and corresponding countermeasures of existing policies or regulations, paying more attention to regional economic development, health system construction, medical service management and so on [19, 27–30]. Few studies have explored the practical impact of cross-regional medical treatment in the context of current policies from a patient perspective, especially their fairness of medical security and individual out-of-pocket expenses. At the same time, the study noted that malignant tumors may be one of the main diseases for cross-regional medical treatment. This is related to the high epidemiological burden of malignant tumors. On the one hand, the overall incidence of malignant tumors in the whole population is increasing. Previous studies have estimated there were 18.1 million new cancer cases worldwide in 2018 alone, and some studies also point out that the global incidence of cancer is expected to increase substantially over the next decades [31, 32]. On the other hand, according to the Global Burden of Disease Study 2019 (GBD 2019), the mortality rate and disability-adjusted life years (DALYs) of malignant tumors both rank first. Such critical diseases are well characterized by planned cross-regional medical treatment. In addition, malignant neoplasms pose a high disease economic burden, especially for patients in low-income areas, who are also an important group for cross-regional medical care [33–38]. Therefore, this study wants to explore the influence of the temporal and spatial characteristics of cross-regional medical treatment on the total medical expenses, medical insurance payments, and individual out-of-pocket expenses of patients with malignant tumors in low-income areas under the background of China’s current health care policies, providing strong evidence for improving the fairness of medical security for cross-regional medical patients and reducing their direct economic burden of disease.

## Methods

### Sample selection and data acquisition

To obtain more samples that meet the research conditions, the insured region should have the characteristics of less developed economy, large outflow population, and higher prevalence of malignant tumors. According to the ranking of per capita gross domestic product (PCGDP) and per capita disposable income (PCDI) of Chinese provinces in 2020 given by the National Bureau of Statistics (NBS), Heilongjiang Province (ranking second from the bottom) was an underdeveloped region with a low income level, and its population outflow was the most serious with the highest population loss rate in China, according to the results of the Seventh National Census. At the same time, Heilongjiang Province is located on the northern border of China, with poor weather conditions, relatively backward medical resources, and higher cancer burden [39, 40]. Therefore, Heilongjiang Province was selected as the research area.

By 2020, Heilongjiang Province had fully realized the direct online reimbursement of hospitalization expenses of authorized cross-provincial patients. For patients without the authorization of medical insurance agencies, they need to return to the insured region for manual reimbursement. For patients without the authorization of medical insurance agencies, they need to return to the insured region for manual reimbursement, whose payment scope is consistent with that of local medical treatment, and the reimbursement ratio is reduced by about 20%. Their medical insurance treatment is naturally very low, and will not change due to the difference in medical treatment regions. This is related to controlling unreasonable medical treatment behavior to maintain the balance of medical insurance funds [41]. With the continuous improvement of the direct settlement system of cross-provincial medical treatment, this kind of population will gradually decrease. At the same time, considering the relatively low cancer survival rate in China [42], cross-sectional studies should be conducted to ensure the timeliness of data and reduce sample mixing. Therefore, this study applied to the Heilongjiang Medical Security Service Center for detailed data of cross-provincial direct settlement of medical expenses of insured malignant tumor patients in Heilongjiang Province in 2020 and promised to protect the privacy of patients, and the data was only used for this study. Finally, 54,904 valid data were obtained.

### Variable selection and setting

In the database used in this study, the expense part includes the total medical expense and its composition, the amount of various medical insurance payments and out-of-pocket expenses of patients. From the perspective of patients, they do not pay attention to the specific composition of medical expenses or the proportion of medical insurance reimbursement stipulated by policies, but pay more attention to the actual level of medicare payments and the personal out-of-pocket amount that can directly affect their economic burden of disease. Therefore, this study selected three indicators as dependent variables: total medical expenses, actual medical insurance payment level (total medicare payments divided by total medical expenses) and personal out-of-pocket expenses. The first two variables were divided into three grades of low, middle and high according to trisection, which not only met the requirements of statistics but also intuitively showed the relative degree of themselves. The third variable referred to the concept of the group with high medical expenditure in the research on the concentration of medical expenditure, and defined the patients with the top 10% of out-of-pocket expenses as the group with high out-of-pocket expenses [43].

The independent variable of this study was the basic attribute of cross-regional medical regions, which was divided into economic levels and geographical distances from the insured region (divided into three grades based on the nationwide rankings), which are the deep-seated factors affecting population mobility and medical level [44, 45]. Since the database contains little personal information about patients in addition to the above expense information, age (divided into the elderly and non-elderly) and insurance type (divided into urban workers and urban and rural residents) which were known to have a greater impact on the dependent variable were selected as control variables in this study [46, 47]. At the same time, one of the independent variables was controlled and the other was analyzed separately to further reduce factor interference.

### Statistical method

This study first applied Microsoft Office Excel 2019 and ArcGIS 10.2 to conduct a descriptive analysis of the temporal and spatial characteristics of the respondents’ cross-provincial medical regions, and then binary and multivariate logistic regression analysis was used to explore respectively the specific impact of economic level and geographical distance of cross-provincial medical regions.

## Results

### Temporal characters of cross-regional medical treatment in patients with malignant tumors

**Fig. 1:**
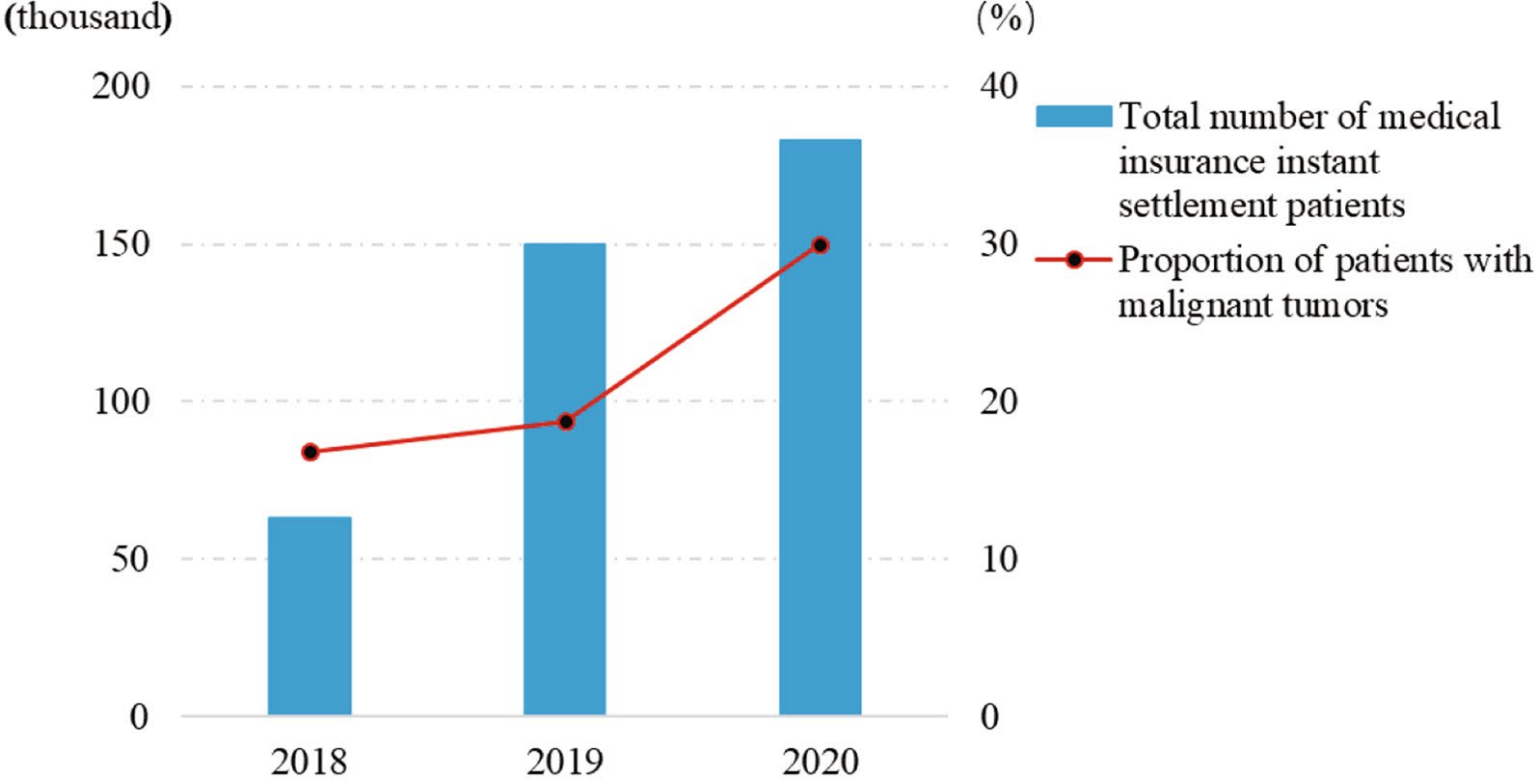
The overall situation of cross-provincial medical patients insured in Heilongjiang Province from 2018 to 2020

**Fig. 2:**
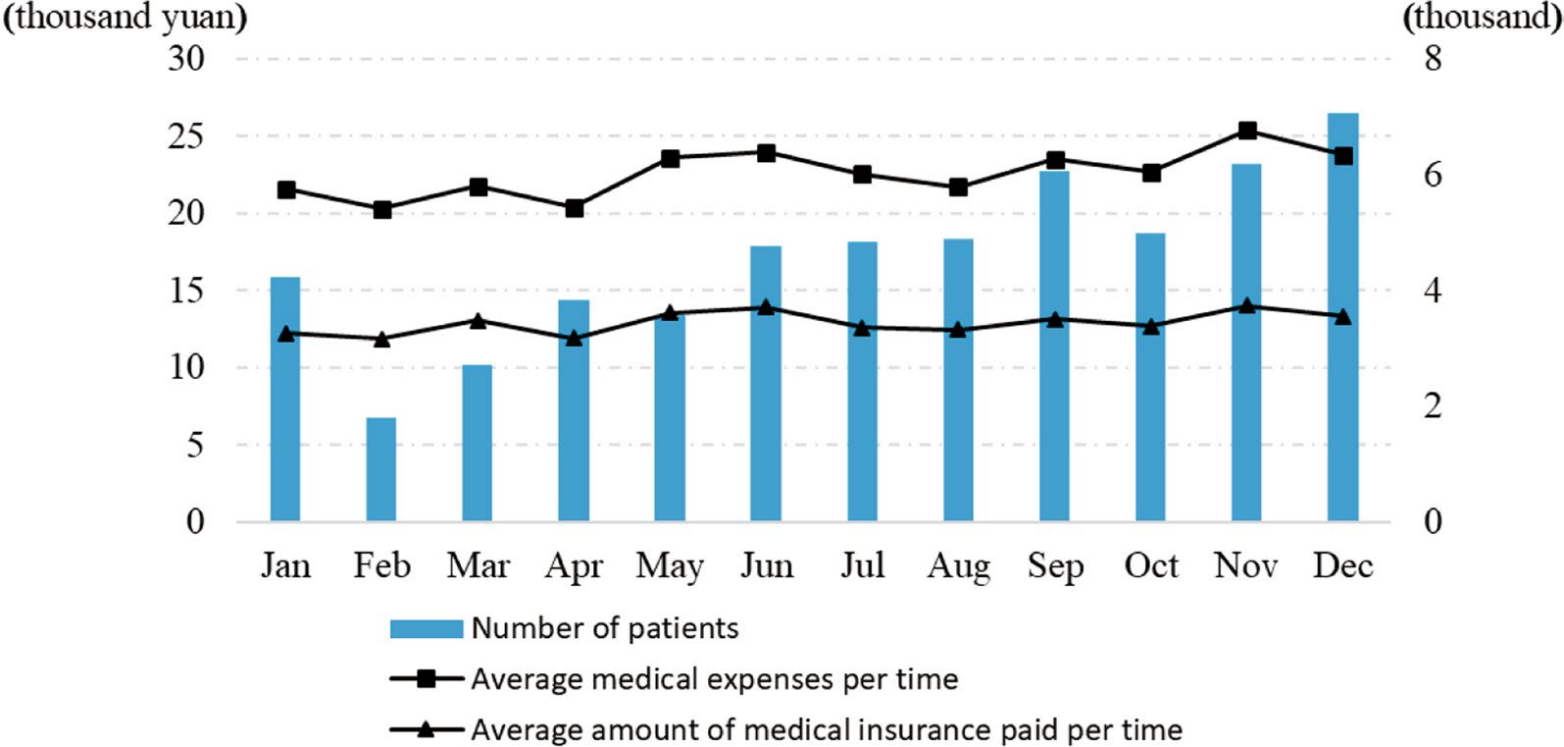
Monthly statistics of cross-provincial medical patients with malignant tumors insured in Heilongjiang Province in 2020

From 2018 to 2020, the total number of instant medical insurance settlements of cross-provincial medical treatment in Heilongjiang Province had kept increasing, among which the proportion of malignant tumor patients had also kept increasing, reaching 30.0% in 2020, an increase of 11.3 percentage points compared with 2019 (Fig. 1). Patients with malignant tumors have become the main group of cross-provincial medical insurance payments. In 2020, the number of them showed a gradual upward trend from February to December, and fell back in January, with a maximum difference of 5253. The variation trend of total medical expense per time was basically the same as that of average medical insurance payment per time, but the former changed more obviously from month to month with a slight increase in general, while the latter was relatively stable (Fig. 2).

### Spatial characters of cross-regional medical treatment in patients with malignant tumors

**Fig. 3:**
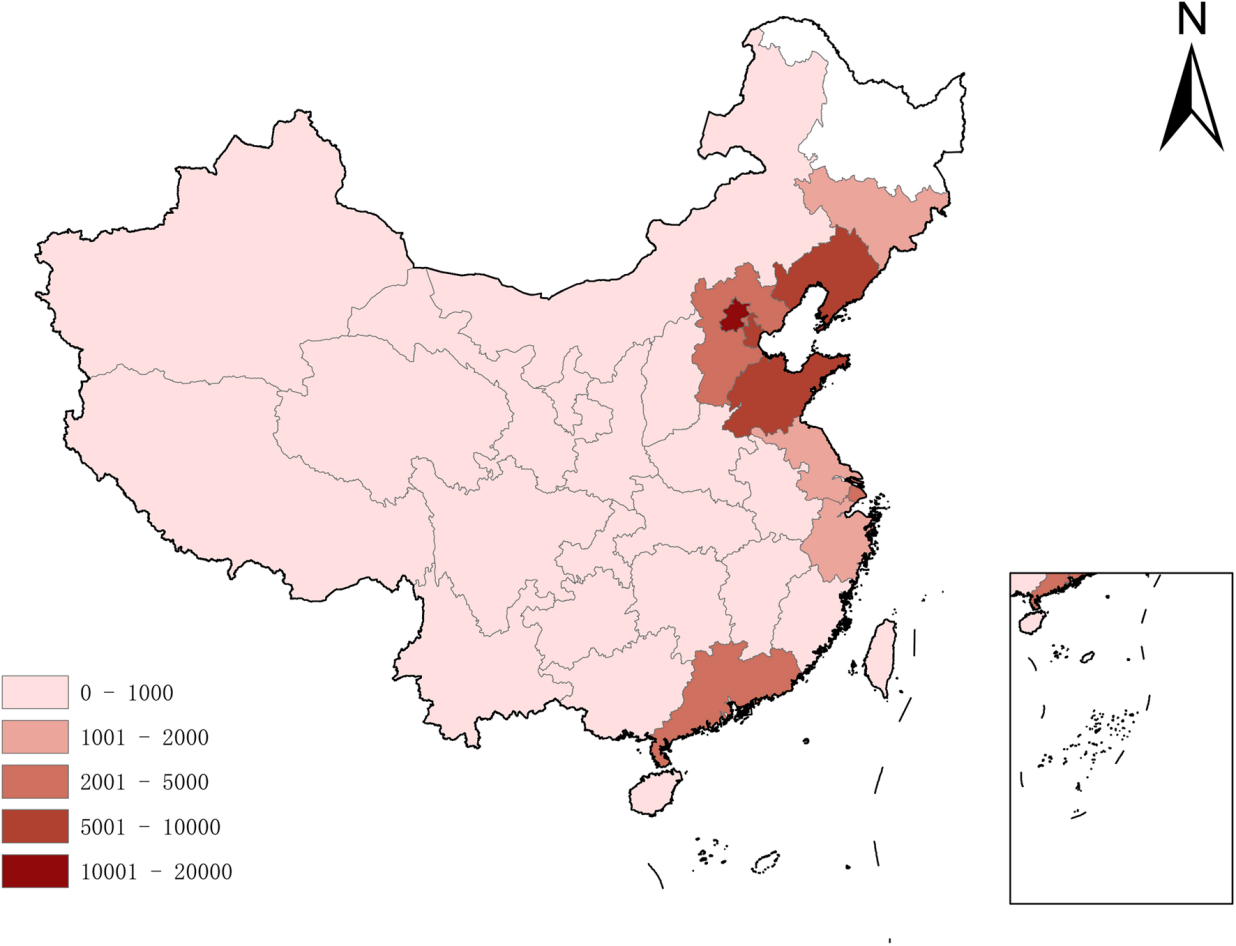
Cross-provincial medical distribution of malignant tumor patients from Heilongjiang Province in 2020

**Fig. 4:**
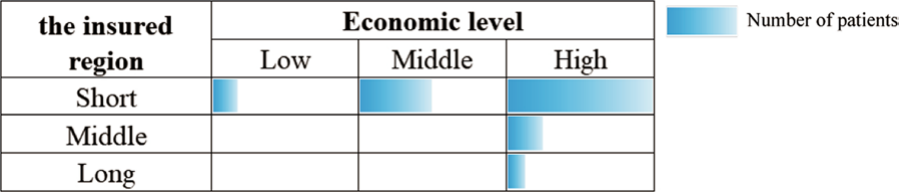
Economic levels and geographical distances distribution of cross-provincial medical regions

In 2020, the cross-provincial medical treatment regions of malignant tumor patients in Heilongjiang Province were mainly distributed in Northeast, North China and southeast coastal areas of China (Fig. 3). In this study, the top 10 regions with the number of patients accounting for 93.3% of total sample were selected to investigate their PCGDPs and distances from insured region among 30 provinces and autonomous regions except Heilongjiang Province and Taiwan Province in China (Table 1), and very 10 rankings were divided into a scale. The results showed that cross-regional medical patients were concentrated in regions with higher economic level and closer to insured region (Fig. 4).

**Table 1:** The top 10 cross-provincial medical regions

Region	N	%	Ranking of PCGDP in 2020 (from high to low)	Ranking of distance from the insured region (from short to long)
Beijing	15,776	28.7	1	5
Tianjin	7960	14.5	5	6
Shandong	6591	12.0	11	7
Liaoning	5974	10.9	15	3
Shanghai	4606	8.4	2	11
Hebei	3053	5.6	27	4
Guangdong	3023	5.5	7	26
Jiangsu	1474	2.7	3	10
Zhejiang	1474	2.7	6	12
Jilin	1300	2.4	24	1
The others	3673	6.7	–	–

### Analysis of the influence of economic levels and distances from the insured region of cross-regional medical regions

In order to control the interaction between independent variables and reduce the confusion, and taking into account the spatial distribution characteristics of samples, this study respectively selected three short-distance medical regions with different economic levels (Beijing, Liaoning and Hebei) and three high economic level medical regions with different distances from the insured region (Tianjin, Zhejiang and Guangdong) to construct an independent logistic regression model. Since the *p* values of the parallel line tests of ordered multivariate logistic regression models without adjustment were all less than 0.01, the models failed the test. This study selected disordered multiple logistic regression models for analysis. According to the adjusted model results: when the distances between cross-regional medical regions and the insured region were basically the same, the patients in the medical region with high economic levels were more likely to form higher total medical expenses (AOR = 2.932), lower actual medical insurance payment level (AOR = 2.603), and the group with high out-of-pocket expenses (AOR = 3.620) (Table 2); When the economic levels of cross-region medical regions were similar, the patients in the short-distance medical region were more likely to form lower actual medical insurance payment level (AOR = 5.976) and the group with high out-of-pocket expenses (AOR = 1.882), while the patients in the middle-distance medical region were less likely to form higher total medical expense (AOR = 0.424) and the group with high out-of-pocket expenses (AOR = 0.545) (Table 3).

**Table 2:** The influence of economic levels of cross-regional medical regions

Variable	N	%	Total medical expenses (reference = low)				Actual medical insurance payment level (reference = high)				The group with high out-of-pocket expenses	
			Middle		High		Low		Middle			
			*P*	*AOR* (95% CI)	*P*	*AOR* (95% CI)	*P*	*AOR* (95% CI)	*P*	*AOR* (95% CI)	*P*	*AOR* (95% CI)
Age												
< 60	9819	39.6	0.909	1.004 (0.940, 1.073)	0.006	1.098* (1.028, 1.174)	< 0.001	1.627* (1.516, 1.745)	< 0.001	1.582* (1.478, 1.693)	0.001	1.157* (1.060, 1.264)
≥ 60 (reference)	14,984	60.4										
Insurance type												
Urban and rural residents	8281	33.4	0.173	0.954 (0.891, 1.021)	0.124	0.947 (0.883, 1.015)	< 0.001	6.138* (5.672, 6.643)	< 0.001	2.656* (2.452, 2.877)	< 0.001	1.377* (1.258, 1.508)
Urban workers (reference)	16,522	66.6										
Economic level												
High	15,776	63.6	< 0.001	1.407* (1.286, 1.539)	< 0.001	2.932* (2.644, 3.250)	< 0.001	2.603* (2.333, 2.904)	< 0.001	1.263* (1.151, 1.385)	< 0.001	3.620* (2.982, 4.394)
Middle	5974	24.1	< 0.001	0.787* (0.712, 0.869)	0.185	1.081 (0.963, 1.212)	< 0.001	3.166* (2.803, 3.577)	< 0.001	1.688* (1.518, 1.878)	< 0.001	2.261* (1.834, 2.788)
Low (reference)	3053	12.3										

This table contained three adjusted logistic regression models and “*” indicated significant at the 0.01 level

**Table 3:** The influence of distances from the insured region

Variable	N	%	Total medical expenses (reference = low)				Actual medical insurance payment level (reference = high)				The group with high out-of-pocket expenses	
			Middle		High		Low		Middle			
			*P*	*AOR* (95% CI)	*P*	*AOR* (95% CI)	*P*	*AOR* (95% CI)	*P*	*AOR* (95% CI)	*P*	*AOR* (95% CI)
Age												
< 60	6761	54.3	< 0.001	1.373* (1.249, 1.510)	< 0.001	1.413* (1.284, 1.554)	< 0.001	1.748* (1.577, 1.937)	< 0.001	1.675* (1.520, 1.845)	0.064	0.886 (0.779, 1.007)
≥ 60 (reference)	5696	45.7										
Insurance type												
Urban and rural residents	4696	37.7	0.255	0.948 (0.865, 1.039)	< 0.001	0.838* (0.764, 0.919)	< 0.001	3.698* (3.334, 4.103)	< 0.001	1.745* (1.575, 1.932)	< 0.001	1.378* (1.220, 1.555)
Urban workers (reference)	7761	62.3										
Distance from the insured region												
Short	7960	63.9	0.378	0.952 (0.853, 1.062)	0.086	0.102 (0.986, 1.231)	< 0.001	5.976* (5.275, 6.771)	< 0.001	3.314* (2.969, 3.698)	< 0.001	1.882* (1.598, 2.217)
Middle	1474	11.8	< 0.001	0.498* (0.429, 0.578)	< 0.001	0.424* (0.362, 0.498)	0.116	0.861 (0.714, 1.038)	0.022	1.184 (1.024, 1.368)	< 0.001	0.545* (0.405, 0.734)
Long (reference)	3020	24.3										

This table contained three adjusted logistic regression models and “*” indicated significant at the 0.01 level

### Sensitivity analysis

This study first conducted sensitivity analysis by adding the covariable length of stay, which may also affect on the dependent variable. The adjusted model results obtained by using the same method were basically unchanged (Tables 4, 5). Subsequently, the patients with lung cancer, which accounted for the largest proportion (23.5%) of the original sample as a single disease, were selected for sensitivity analysis excluding the interference of disease species. The new sample was used to repeat the above modeling steps of this study, and the adjusted model results were basically consistent with the previous analysis results of the full sample, which indicated once again that the results of this study were relatively stable and reliable (Additional file 1: Tables S1, S2).

**Table 4:** Sensitivity analysis of the models in Table 2

Variable	M/N	(P25, P75)/%	Total medical expenses (reference = low)				Actual medical insurance payment level (reference = high)				The group with high out-of-pocket expenses	
			Middle		High		Low		Middle			
			*P*	*AOR* (95% CI)	*P*	*AOR* (95% CI)	*P*	*AOR* (95% CI)	*P*	*AOR* (95% CI)	*P*	*AOR* (95% CI)
Length of stay	5	(2, 11)	< 0.001	1.251* (1.238, 1.263)	< 0.001	1.506* (1.490,1.523)	< 0.001	0.988* (0.985, 0.992)	< 0.001	1.006* (1.003, 1.009)	< 0.001	1.106* (1.101, 1.110)
Age												
< 60	9819	39.6	0.010	1.097 (1.022, 1.176)	< 0.001	1.412* (1.295, 1.540)	< 0.001	1.619* (1.508, 1.737)	< 0.001	1.584* (1.480, 1.695)	< 0.001	1.244* (1.127, 1.374)
≥ 60 (reference)	14,984	60.4										
Insurance type												
Urban and rural residents	8281	33.4	0.006	0.902* (0.839, 0.970)	< 0.001	0.801* (0.731, 0.877)	< 0.001	6.183* (5.713, 6.692)	< 0.001	2.649* (2.446, 2.869)	< 0.001	1.440* (1.301, 1.593)
Urban workers (reference)	16,522	66.6										
Economic level												
High	15,776	63.6	< 0.001	2.608* (2.357, 2.887)	< 0.001	15.509* (13.274, 18.120)	< 0.001	2.551* (2.286, 2.847)	< 0.001	1.279* (1.166, 1.404)	< 0.001	8.375* (6.576, 10.667)
Middle	5974	24.1	0.070	0.905 (0.813, 1.008)	< 0.001	2.129* (1.087, 2.510)	< 0.001	3.109* (2.752, 3.513)	< 0.001	1.713* (1.540, 1.905)	< 0.001	5.159* (3.994, 6.662)
Low (reference)	3053	12.3										

This table contained the results of three models after adding the length of stay variable and “*” indicated significant at the 0.01 level

**Table 5:** Sensitivity analysis of the models in Table 3

Variable	M/N	(P25, P75)/%	Total medical expenses (reference = low)				Actual medical insurance payment level (reference = high)				The group with high out-of-pocket expenses	
			Middle		High		Low		Middle			
			*P*	*AOR* (95% CI)	*P*	*AOR* (95% CI)	*P*	*AOR* (95% CI)	*P*	*AOR* (95% CI)	*P*	*AOR* (95% CI)
Length of stay	5	(2, 10)	< 0.001	1.247* (1.228, 1.268)	< 0.001	1.593* (1.564, 1.622)	< 0.001	0.997* (0.992, 1.002)	< 0.001	1.010* (1.006, 1.015)	< 0.001	1.113* (1.106, 1.119)
Age												
< 60	6761	54.3	< 0.001	1.596* (1.445, 1.764)	< 0.001	2.278* (2.002, 2.592)	0.274	1.742 (1.572, 1.931)	< 0.001	1.684* (1.528, 1.856)	0.217	0.913 (0.791, 1.055)
≥ 60 (reference)	5696	45.7										
Insurance type												
Urban and rural residents	4696	37.7	0.003	0.866* (0.787, 0.953)	< 0.001	0.627* (0.554, 0.709)	< 0.001	3.707* (3.341, 4.113)	< 0.001	1.735* (1.567, 1.922)	< 0.001	1.392* (1.214, 1.595)
Urban workers (reference)	7761	62.3										
Distance from the insured region												
Short	7960	63.9	0.664	1.027 (0.916, 1.152)	< 0.001	1.517* (1.305, 1.763)	< 0.001	5.971* (5.270, 6.766)	< 0.001	3.346* (2.997, 3.735)	0.003	2.574* (2.132, 3.108)
Middle	1474	11.8	< 0.001	0.603* (0.515, 0.705)	< 0.001	0.554* (0.441, 0.697)	0.016	0.857 (0.710, 1.033)	0.010	1.210 (1.047, 1.400)	< 0.001	0.576* (0.403, 0.824)
Long (reference)	3023	24.3										

This table contained the results of three models after adding the length of stay variable and “*” indicated significant at the 0.01 level

## Discussion

### There are seasonal differences in cross-regional medical treatment

According to the temporal characteristics of cross-regional medical treatment in patients with malignant tumors, there were certain seasonal differences in cross-regional medical treatment. This may be related to seasonal characteristics of population mobility and disease incidence [48, 49].

In terms of the number of patients, one year can be divided into four stages: falling period, rapid growth period, transition period, and peak period. Firstly, due to the custom of returning home during the Spring Festival in China, a large number of people temporarily living in other regions went back to their hometowns during the Spring Festival in January and February [50], which may lead to a large decrease in the number of unplanned cross-regional medical patients. After the Spring Festival, people returned to the regions where they used to live due to work, study, and other reasons, forming the return peak, meanwhile, due to the rapid warming of the southern region since March, rural migrant workers in the north gradually left their homes and began to resume work [51, 52]. This would lead to a rapid increase in the number of cross-regional patients in spring (from Feb to Apr). In summer and autumn (from May to Sept), due to more frequent travels, business trips, and other outings, the probability of emergency treatment, hospitalization, and other unplanned medical behaviors in other regions caused by emergencies would increase [53, 54], which would lead to a slight increase in the total number of cross-regional medical patients. Then, the winter in Heilongjiang Province had started from October with a sudden drop in temperature [39, 55]. Although the number of people going out mentioned above would decrease affected by the weather, the long heating season can also lead to a decline in air quality due to a large number of air pollutant emissions [56], which can increase the risk of cancer incidence [57], meanwhile, the harsh climatic conditions in winter would bring some challenges to the sustainable treatment of cancer [58], in addition to the fact that the backward medical resources in the insured region cannot meet the needs of patients for accurate diagnosis and high-quality medical care. Therefore, the planned cross-regional medical treatment of malignant tumor patients increased significantly, resulting in the total number of patients decreased first and then rose rapidly from October to December, and reached the peak at the end of the year.

As can be seen from Fig. 2, although the number of patients was constantly changing, seasonal factors had little influence on the average medical expense per time and the average medical insurance payment amount per time. The monthly fluctuation trends of the two were basically the same, but the fluctuation range of the former was slightly larger, and it generally increased in winter. This phenomenon showed that the medical insurance payment did not respond well to the changes of medical expenses, especially in winter, when the increase of severe diseases may lead to higher treatment expenses, the medical insurance policies had not been timely adjusted, which was likely to increase in the average out-of-pocket expenses of cross-regional patients with malignant tumors.

### The patients in cross-regional medical regions with high economic level are more likely to form the group with high out-of-pocket expenses

This survey found that the number of cross-regional medical patients in regions with high economic level accounted for more than 60% of the total sample, and nearly 60% of the short-distance cross-regional medical treatment occurred in areas with high economic level (Table 1). This is because higher economic levels often mean higher income, more employment opportunities, and better medical conditions, which can attract more floating population (unable to obtain household register) and out-of-town patients [37, 44, 59]. However, for patients with malignant tumors, such regions were more likely to form higher total medical expenses and lower actual medical insurance payment levels, thus increasing the possibility of becoming the group with high out-of-pocket expenses (Table 2). This should be the result of the combined influence of the regional economy, medical insurance policies and population mobility. On the one hand, under the influence of the market economy, the higher the regional economic level, the higher the price of medical service, and the threshold line for medical insurance may be [60]. And hospitals with better medical conditions tend to use more high-end medical devices and innovative drugs that are not covered by health insurance or have lower reimbursement rates [61, 62]. If the reimbursement level specified by the policy remains unchanged, the actual payment level will decrease and the out-of-pocket expenses of patients will rise significantly. On the other hand, compared with low-income areas, high-income areas can attract more young labor force and increase the proportion of non-elderly people in unplanned cross-regional medical patients [63]. When they suffer from malignant tumors, they tend to choose more advanced treatments for longer survival times, which makes them likely to have longer treatment cycles, less probability of palliative care, and higher medical expenses than the elderly [64, 65], in addition, the medicare treatment of the elderly in China is generally higher. All of these can make non-elderly people more likely to become patients with high out-of-pocket expenses, and also increase the probability of patients with high out-of-pocket expenses in regions with high economic levels (Table 2).

### The middle-distance medical regions may be the best choice for patients with planned cross-regional medical treatment

More than 70% of the medical regions with a high economic level in this survey were close to the insured regions (Table 1). This indicated that the principle of proximity existed in cross-regional medical treatment. Especially for patients with planned cross-regional medical treatment, while pursuing high-quality medical services, most of them tend to choose short-distance cross-regional medical regions due to the consideration of medical urgency and transportation, accommodation and other non-medical economic burdens [6]. However, the results of this study showed that the possibility of becoming patients with high out-of-pocket expenses was less in the middle-distance medical regions (Table 3). This can also be explained in terms of medical insurance policies and population mobility. On the one hand, to control a large number of unreasonable referrals and relieve the payment pressure of local medical insurance funds, the reimbursement ratios of planned cross-regional medical treatment are often reduced by local governments [66]. This approach may be radical and to some extent undermines the rights of patients who are normally referred. On the other hand, as winter weather conditions in South China are more suitable for living or recuperating, a large number of “migratory birds” elderly have appeared in northeast China in recent years, and they are concentrated in Guangdong, Hainan and its surrounding areas, resulting in a significant increase in long-distance cross-regional medical treatment [67, 68]. In response, Heilongjiang Province has introduced a policy of exemption from authorization to ensure the normal reimbursement of the “migratory birds” elderly for medical treatment (http://ybj.hlj.gov.cn/ljyb/1849.jhtml). However, this study found that the average length of stay for such long-distance medical treatment was longer, increasing the risk of high medical costs (Table 5), which may be related to the higher expenditures on drugs and medical devices [68], as well as a lack of remote collaborative supervision resulting in excessive diagnosis and treatment.

## Limitations

In this study, some limitations are present. Although the sample size of this study is large, there are fewer covariables in this study due to less basic information of patients available in the database, which may affect the credibility of the model results. In addition, this is a cross-sectional study and it is difficult to establish a causal relationship between variables. Future research may use panel data with more variables and do further analysis on patients with specific diseases.

## Conclusion

Cross-regional medical patients with malignant tumors in low-income areas are concentrated in the regions with higher economic level and closer to their insured regions, the number of them will be affected by seasonal factors. At the same time, their total medical expenses, actual medical insurance payment levels and out-of-pocket expenses are all affected by the economic levels and distances of medical regions. And the middle-distance medical regions may be the best choice for patients with planned cross-regional medical treatment. The relevant policymakers should pay attention to the above phenomena, fully consider patient mobility and regional differences, and reasonably improve the fairness, responsiveness and sustainability of medical insurance for cross-regional medical patients with serious diseases so as to further reduce their out-of-pocket expense burden. At the same time, more attention should be paid to the supervision of cross-regional medical treatment and the development of telemedicine.

## Supplementary Information

**Additional file 1: Table S1.** The second sensitivity analysis of the models in Table 2. Table S2. The second sensitivity analysis of the models in Table 3.

## Data Availability

The datasets generated and analyzed during the current study are not publicly available because the datasets are currently used for another project, but are available from the corresponding author on reasonable request.

## Abbreviations

NHS: National Health Service
EU: European Union
EHIC: European Health Insurance Card
EC: European Community
GBD: Global Burden of Disease
DALY: Disability-adjusted life year
PCGDP: Per capita gross domestic product
PCDI: Per capita disposable income
NBS: National Bureau of Statistics
