# Supplementary material for: The influence of cross-regional medical treatment on total medical expenses, medical insurance payments, and out-of-pocket expenses of patients with malignant tumors in Chinese low-income areas
Source: Cost Eff Resour Alloc. 2022 Jul 21;20:35. doi: 10.1186/s12962-022-00368-x (PMC9306213; doi:10.1186/s12962-022-00368-x)
Supplement: Supplementary file 1 — Additional file 1: Table S1. The second sensitivity analysis of the models in Table 2. Table S2. The second sensitivity analysis of the models in Table 3. [file 12962_2022_368_MOESM1_ESM.docx]

**Table S1. The second sensitivity analysis of the models in Table 2**

| **Variable** | **N** | **%** | **Total medical expenses (Reference = Low)** | | | | **Actual medical insurance payment level**  **(Reference = High)** | | | | **The group with high out-of-pocket expenses** | |
| --- | --- | --- | --- | --- | --- | --- | --- | --- | --- | --- | --- | --- |
|  |  |  | **Middle** | | **High** | | **Low** | | **Middle** | |  |  |
|  |  |  | ***P*** | ***AOR* (95%*CI*)** | ***P*** | ***AOR* (95%*CI*)** | ***P*** | ***AOR***  **(95%*CI*)** | ***P*** | ***AOR* (95%*CI*)** | ***P*** | ***AOR***  **(95%*CI*)** |
| **Age** |  |  |  |  |  |  |  |  |  |  |  |  |
| < 60 | 2061 | 29.8 | 0.427 | 0.948  (0.831,1.082) | 0.200 | 1.089  (0.956,1.242) | <0.001 | 2.177^*^  (1.886,2.511) | <0.001 | 1.634^*^  (1.419,1.881) | <0.001 | 1.574^*^  (1.330,1.862) |
| ≥ 60 (Reference) | 4859 | 70.2 |  |  |  |  |  |  |  |  |  |  |
| **Insurance type** |  |  |  |  |  |  |  |  |  |  |  |  |
|  |  |  |  |  |  |  |  |  |  |  |  |  |
| Urban and rural residents | 2054 | 29.7 | 0.830 | 0.986  (0.866,1.122) | 0.062 | 0.882  (0.772,1.006) | <0.001 | 6.410^*^  (5.481,7.496) | <0.001 | 2.972^*^  (2.535,3.484) | 0.006 | 1.269^*^  (1.069,1.506) |
| Urban workers (Reference) | 4866 | 70.3 |  |  |  |  |  |  |  |  |  |  |
|  |  |  |  |  |  |  |  |  |  |  |  |  |
| **Economic level** |  |  |  |  |  |  |  |  |  |  |  |  |
| High | 5424 | 78.4 | 0.002 | 1.362^*^  (1.118,1.659) | <0.001 | 3.836^*^  (2.944,4.998) | <0.001 | 2.533^*^  (1.967,3.262) | 0.001 | 1.434^*^  (1.160,1.7740 | <0.001 | 5.195^*^  (2.837,9.515) |
| Middle | 963 | 13.9 | 0.155 | 0.841  (0.663,1.068) | <0.001 | 1.977^*^  (1.466,2.666) | <0.001 | 7.154^*^  (5.281,9.690) | <0.001 | 2.950^*^  (2.248,3.387) | <0.001 | 8.126^*^  (4.352,15.172) |
| Low (Reference) | 533 | 7.7 |  |  |  |  |  |  |  |  |  |  |

**Note:** This table contained the results of three models based on samples of lung cancer patients and "*" indicated significant at the 0.01 level

**Table S2. The second sensitivity analysis of the models in Table 3**

| **Variable** | **N** | **%** | **Total medical expenses (Reference = Low)** | | | | **Actual medical insurance payment level**  **(Reference = High)** | | | | **The group with high out-of-pocket expenses** | |
| --- | --- | --- | --- | --- | --- | --- | --- | --- | --- | --- | --- | --- |
|  |  |  | **Middle** | | **High** | | **Low** | | **Middle** | |  |  |
|  |  |  | ***P*** | ***AOR* (95%*CI*)** | ***P*** | ***AOR* (95%*CI*)** | ***P*** | ***AOR***  **(95%*CI*)** | ***P*** | ***AOR* (95%*CI*)** | ***P*** | ***AOR* (95%*CI*)** |
| **Age** |  |  |  |  |  |  |  |  |  |  |  |  |
| < 60 | 675 | 35.7 | 0.251 | 1.158  (0.901,1.488) | 0.105 | 1.231  (0.958,1.582) | <0.001 | 1.891^*^  (1.413,2.530) | <0.001 | 1.644^*^  (1.248,2.166) | 0.012 | 1.513  (1.097,2.087) |
| ≥ 60 (Reference) | 1214 | 64.3 |  |  |  |  |  |  |  |  |  |  |
| **Insurance type** |  |  |  |  |  |  |  |  |  |  |  |  |
| Urban and rural residents | 597 | 31.6 | 0.857 | 1.023  (0.802,1.304) | 0.315 | 0.880  (0.687,1.129) | <0.001 | 8.729^*^  (6.383,11.938) | <0.001 | 3.220^*^  (2.357,4.400) | 0.004 | 1.584^*^  (1.158,2.168) |
| Urban workers (Reference) | 1292 | 68.4 |  |  |  |  |  |  |  |  |  |  |
| **Distance from the insured**  **region** |  |  |  |  |  |  |  |  |  |  |  |  |
| Short | 955 | 50.6 | 0.035 | 1.325  (1.020,1.722) | <0.001 | 1.830^*^  (1.404,2.386) | <0.001 | 7.815^*^  (5.735,10.650) | <0.001 | 5.221^*^  (3.934,6.929) | <0.001 | 3.039^*^  (2.001,4.615) |
| Middle | 290 | 15.4 | <0.001 | 0.485^*^  (0.349,0.675) | <0.001 | 0.423^*^  (0.294,0.607) | 0.958 | 0.989  (0.665,1.472) | 0.438 | 1.140  (0.818,1.589) | 0.077 | 0.471  (0.205,1.085) |
| Long (Reference) | 644 | 34.1 |  |  |  |  |  |  |  |  |  |  |

**Note:** This table contained the results of three models based on samples of lung cancer patients and "*" indicated significant at the 0.01 level
